# Supplementary material for: Causal Relationship between Aging and Anorexia Nervosa: A White-Matter-Microstructure-Mediated Mendelian Randomization Analysis
Source: Biomedicines. 2024 Aug 16;12(8):1874. doi: 10.3390/biomedicines12081874 (PMC11351342; doi:10.3390/biomedicines12081874)
Supplement: Supplementary file 1 [file biomedicines-12-01874-s001.zip › Supplementary Figure/Figure S4/Singe SNP plot of MR analysis for TL to ubm-b-1495.pdf]

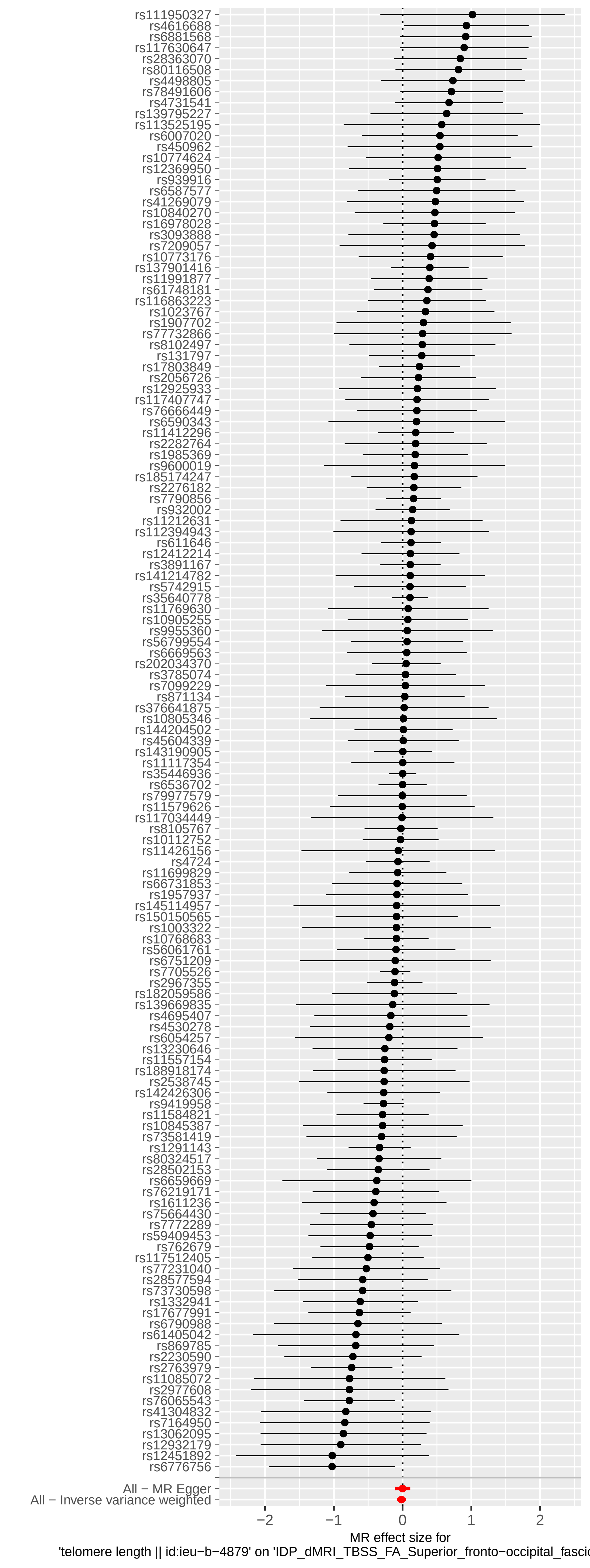
rs111950327
rs4616688
rs6881568
rs117630647
rs28363070
rs80116508
rs4498805
rs78491606
rs4731541
rs139795227
rs113525195
rs6007020
rs450962
rs10774624
rs12369950
rs939916
rs6587577
rs41269079
rs10840270
rs16978028
rs3093888
rs7209057
rs10773176
rs137901416
rs11991877
rs61748181
rs116863223
rs1023767
rs1907702
rs77732866
rs8102497
rs131797
rs17803849
rs2056726
rs12925933
rs117407747
rs76666449
rs6590343
rs11412296
rs2282764
rs1985369
rs9600019
rs185174247
rs2276182
rs7790856
rs932002
rs11212631
rs112394943
rs611646
rs12412214
rs3891167
rs141214782
rs5742915
rs35640778
rs11769630
rs10905255
rs9955360
rs56799554
rs6669563
rs202034370
rs3785074
rs7099229
rs871134
rs376641875
rs10805346
rs144204502
rs45604339
rs143190905
rs11117354
rs35446936
rs6536702
rs79977579
rs11579626
rs117034449
rs8105767
rs10112752
rs11426156
rs4724
rs11699829
rs66731853
rs1957937
rs145114957
rs150150565
rs1003322
rs10768683
rs56061761
rs6751209
rs7705526
rs2967355
rs182059586
rs139669835
rs4695407
rs4530278
rs6054257
rs13230646
rs11557154
rs188918174
rs2538745
rs142426306
rs9419958
rs11584821
rs10845387
rs73581419
rs1291143
rs80324517
rs28502153
rs6659669
rs76219171
rs1611236
rs75664430
rs7772289
rs59409453
rs762679
rs117512405
rs77231040
rs28577594
rs73730598
rs1332941
rs17677991
rs6790988
rs61405042
rs869785
rs2230590
rs2763979
rs11085072
rs2977608
rs76065543
rs41304832
rs7164950
rs13062095
rs12932179
rs12451892
rs6776756
All − MR Egger
All − Inverse variance weighted
−2
−1
0
1
2
MR effect size for
'telomere length || id:ieu−b−4879' on 'IDP_dMRI_TBSS_FA_Superior_fronto−occipital_fasci
